# Initiation of the expression of peroxisome proliferator - activated receptor gamma (PPAR gamma) in the rat ovary and the role of FSH

**DOI:** 10.1186/1477-7827-7-145

**Published:** 2009-12-07

**Authors:** Mary J Long, M Ram Sairam, Carolyn M Komar

**Affiliations:** 1 Department of Animal Science, Iowa State University, Ames, IA 50011, USA; 2 Molecular Reproduction Research Laboratory, Institut de Recherches Cliniques de Montreal Montreal, Quebec, H2W 1R7, Canada; 3 Department of Biomedical Sciences, West Virginia School of Osteopathic Medicine, Lewisburg, WV 24901, USA

## Abstract

PPARgamma is highly expressed in granulosa cells by 23 days post-partum (pp) and is down-regulated in response to the LH surge. We tested the hypothesis that high levels of FSH during the neonatal period trigger the expression of PPARgamma. To determine when PPARgamma expression is initiated, ovaries were collected from neonatal rats. Messenger RNA for PPARgamma was undetectable on day 1, low from days 5-14, and increased by day 19 pp (p < 0.05). PPARgamma was detected in select granulosa cells in primary/early secondary follicles. Messenger RNA for the FSH receptor was detected as early as day 1 and remained steady throughout day 19 pp. The FSH receptor was detected by immunoblot analysis in ovaries collected 1, 2, and 5-9 days pp. In a subsequent experiment, neonatal rats were treated with acyline (GnRH antagonist) which significantly reduced FSH (p < 0.05) but not levels of mRNA for PPARgamma. The role of FSH in the induction of PPARgamma expression was further assessed in ovarian tissue from FORKO mice. Both mRNA and protein for PPARgamma were identified in ovarian tissue from FORKO mice. In summary, the FSH/FSH receptor system is present in granulosa cells prior to the onset of expression of PPARgamma. Reducing FSH during the neonatal period, or the ability to respond to FSH, did not decrease expression of mRNA for PPARgamma. These data indicate that FSH is not a primary factor initiating the expression of PPARgamma and that other agents play a role in activating its expression in the ovary.

## Background

Peroxisome proliferator-activated receptor γ (PPARγ) is a member of the steroid receptor superfamily. This transcription factor heterodimerizes with the 9, *cis*-retinoic acid receptor (RXR) and binds to a short sequence of DNA, a PPAR response element (PPRE), present in the promoter region of target genes. PPARγ is activated by a variety of factors such as fatty acids, non-steroidal anti-inflammatory drugs (see [1] for a review), prostaglandins [1-4], oxidized products of LDL (9-HODE and 13-HODE; see [2] for a review), and thiazolidinediones (TZDs) [5,6].

TZDs are a family of drugs which are insulin-sensitizers and agonists of PPARγ. They are used to treat people with type II diabetes. Several studies have demonstrated that TZDs are effective therapeutic agents for some women with polycystic ovary syndrome (PCOS), a leading cause of infertility in premenopausal women [7]. Clinically, PCOS is characterized by hyperandrogenism, anovulation, and frequently the women are insulin resistant. Administration of troglitazone to women with PCOS reduced androgen levels, improved hyperinsulinemia, and in some women restored ovulation [8-11]. Recent studies have also shown that administration of rosiglitazone [12] or pioglitazone [13] to women with PCOS restored ovulation (reviewed in [14]).

Initially, PPARγ was identified as an adipocyte differentiation factor (reviewed in [15]). It has since been shown to be involved in a variety of physiological processes, many of which impact ovarian function. For example, activation of PPARγ influences the production of estradiol, progesterone, and prostaglandins (reviewed in [14,16,17]). It can also regulate the expression of plasminogen activators and matrix metalloproteinases (reviewed in [16]), proteolytic enzymes involved in ovarian tissue remodeling and angiogenesis [18-20].

The expression of PPARγ has been identified in ovarian tissue from a variety of species: humans [21], cattle [22,23], sheep [24], pigs [25], hamsters [26], rats [27,28], and mice [29]. In addition, Mohan *et al*. 2002 reported that PPARγ is also expressed in bovine oocytes [30]. Previous work from our laboratory and others has shown that PPARγ is expressed primarily in granulosa cells of developing follicles [24,28]. The expression of PPARγ is lower in follicles expressing the LH receptor compared to those that do not express the LH receptor [31], and expression of PPARγ is dramatically down-regulated in response to the LH surge [28]. It is not known however, what stimulates the expression of this transcription factor in granulosa cells, nor at what stage of follicular development expression of PPARγ is initiated.

Since PPARγ is activated by drugs in clinical use and dietary factors, and can impact various processes critical for normal ovarian function, it is important to gain a better understanding of how PPARγ is regulated in the ovary. The following experiments were conducted to determine when the expression of PPARγ is initiated in granulosa cells and to test the hypothesis that its expression is stimulated by FSH.

## Methods

### Animals

All procedures involving animals were approved prior to use by the Iowa State University or the Institut de Recherches Cliniques de Montreal Institutional Animal Care and Use Committee. Animals were housed in a controlled environment with a 14:10 light:dark cycle and had free access to food and water. Pregnant Sprague-Dawley rats were monitored daily for the delivery of pups with the day of birth = day 0 post partum (pp). Chemicals and reagents were obtained from Sigma Aldrich (St. Louis, MO, USA) unless specified otherwise.

To determine when the expression of PPARγ was initiated in ovarian cells, ovaries were collected from neonatal rat pups on days 1, 5, 7, 9, 11, 14, and 19 pp. Tissues were frozen at -80°C or fixed in 4% paraformaldehyde. Frozen tissues were processed for the isolation of RNA or protein; fixed tissues were embedded in paraffin for immunohistochemical analysis.

The effect of FSH on levels of mRNA for PPARγ were studied in both neonatal and immature, juvenile rats. Neonatal animals were treated subcutaneously, daily from days 1-7 pp with vehicle (5% (v/v) mannitol in water; n = 7) or the GnRH antagonist, acyline (100 μg/day; n = 6; kindly provided by Dr. R. Blye, Center for Population Research, NICHD). On day 8 pp, ovaries and serum were collected and frozen for later analysis by RT-PCR or RIA, respectively.

Ovaries were also collected from immature rats after priming with estradiol. Twenty-three day old rats were treated with estradiol (1.5 mg/day in corn oil), subcutaneously, for three days. Granulosa cells were collected and 500,000 cells/well cultured as described previously [28] with the following modifications. Cells were cultured in duplicate with or without FSH (50 ng/ml) for 4 or 24 hours (n = 3 experiments). At the end of culture, granulosa cells were subjected to a direct lysate RNase protection analysis.

The role of FSH in initiating the expression of PPARγ was further investigated by analyzing the levels of mRNA and the expression of protein for PPARγ in ovarian tissue from follitropin receptor knock out (FORKO) mice. Ovaries were collected from mice carrying no alleles (-/-), one allele (+/-) or both alleles (+/+) for the FSH receptor at 3 weeks and 3 months of age (n = 3 animals/genotype/age group). Tissues were placed in RNA Later (Ambion, Austin, TX, USA) until processed for RNA and protein isolation. Protein and mRNA for PPARγ was analyzed by western immunoblot and reverse transcriptase (RT)-PCR, respectively.

### Immunohistochemistry

PPARγ was immunolocalized in ovarian tissue collected from neonatal rats at defined times pp as described previously [32]. Paraffin embedded ovarian tissues were serially sectioned at 5 μm. Tissues were processed using an anti-PPARγ antibody (E-8, Santa Cruz Biotechnology, Santa Cruz, CA, USA). Normal goat serum (Vector Labs, Berlingame, CA, USA) was used in place of the primary antibody as a control. Serial sections from each of 3-4 animals/time point were analyzed. A minimum of 3 sections/animal/time point were analyzed.

### Semi-quantitative reverse transcriptase (RT)-PCR

Total RNA was extracted from ovarian tissue collected from FORKO mice and neonatal rats treated with vehicle or acyline, with TRIZOL reagent according to the manufacture's instructions. Complementary DNA was synthesized from 1-2 μg of total RNA with Oligo dT as recommended for SuperScript II reverse transcriptase. Following quantification of cDNA by spectrophotometry, the transcript for PPARγ and the housekeeping gene, S16, were amplified by PCR. Primers used for PPARγ [33] and S16 [34] were published previously. Each reaction amplifying PPARγ and S16 consisted of 1 μM of each primer, 1.25 mM MgCl_2_, 200 μM dNTPs, 1× PCR buffer, 10× bovine serum albumin, and 2 U Taq-polymerase. Concentrations of cDNA used for amplification were 100 ng/reaction for PPARγ and 10 ng/reaction for S16. Reaction conditions were as follows: 35 cycles at 95°C for 2 minutes, 95°C for 1 minute, and 52°C for 1 minute, 72°C for 1 minute, 72°C for 5 minutes. Amplified products were separated by gel electrophoresis through 2% agarose gels containing ethidium bromide. Densitometry was performed using Alpha Innotech SpotDenso software and levels of mRNA for PPARγ were standardized to levels of mRNA for S16/sample. All reagents used for semi-quantitaive RT-PCR were purchased from Invitrogen (Carlsbad, CA, USA), with the exception of the Taq-polymerase (Bioline, Randolph, MA, USA). Primers were synthesized by Integrated DNA Technologies (Coralville, IA, USA).

To ensure the semi-quantitative nature of the assay, the number of cycles per program and the amount of cDNA used as a starting template were tested. The number of cycles used in each PCR program was selected from within the range that yielded output in a linear relationship to input as determined by densitometry. Similarly, the amount of cDNA used was determined by selecting from within the linear range of output paralleling changing concentrations of cDNA in the reaction.

### Western immunoblot

Total protein was isolated from ovarian tissues collected at defined times pp (n = 3-4 animals/day) as described previously [32] and from FORKO mice using TRIZOL reagent according to the manufacturer's instructions. Immunoblot analysis was conducted as described previously [32]. Briefly, protein (5 μg) was separated on a 10% polyacrylamide gel and transferred to a nitrocellulose membrane (Amersham/GE Healthcare, Piscataway, NJ, USA). The membrane was processed with a goat anti-human FSH receptor antibody (Santa Cruz). Membranes were subsequently stripped of conjugates with a β-mercaptoethanol buffer (100 mM β-mercaptoethanol in TBST, 30 min, 42°C with rocking) and re-probed with an antibody against β-actin (Biomol, Plymouth Meeting, PA). Membranes were exposed to Kodak X-OMAT autoradiography film.

### Ribonuclease protection assays (RPA)

Total RNA was isolated from ovarian tissues collected from neonatal animals at defined time points pp using Trizol reagent according to manufacturer's instructions. Levels of mRNA for PPARγ, the FSH receptor, and the ribosomal protein, L32, were measured by RPA as described previously [28] using reagents from Ambion. Rat cDNA for the FSH receptor (plasmid containing the cDNA kindly provided by Dr. Kelly Mayo, Northwestern University, Evanston, IL), was linearized with *BseR1 *and transcribed using ^[α-32P]^CTP and T3 RNA polymerase. Samples of RNA were hybridized overnight with excess radiolabeled antisense riboprobes for PPARγ, the FSH receptor, and L32. Protected fragments were analyzed by polyacrylamide gel electrophoresis. Relative levels of mRNA for PPARγ, the FSH receptor, and L32 were quantified using a phosphor-imager (Molecular Dynamics, Inc., Sunnyvale, CA, USA). The band intensity of mRNA for PPARγ and the FSH receptor was normalized to the corresponding band for L32 per sample.

Lysate RPAs were conducted as described previously [32,35] to measure levels of mRNA for PPARγ in granulosa cells after culture with or without FSH. Granulosa cell lysates were processed and levels of mRNA measured as described above using reagents from Ambion.

### Radioimmunoassay

The concentration of FSH was determined in serum collected from neonatal animals on day 8 pp after treatment with water or acyline. Samples were sent to A. F. Parlow at the National Hormone and Peptide Program (Torrance, CA) and analyzed by RIA. Data are presented as means ± SEM.

### Statistical analysis

Differences in levels of mRNA for PPARγ and the FSH receptor in rat ovarian tissues, and concentrations of FSH were analyzed by ANOVA. Data from the study of mRNA for PPARγ in ovaries from FORKO animals were log transformed prior to analysis by ANOVA. Post-hoc comparisons were made with Tukey's HSD test. A p < 0.05 denoted significant differences.

## Results

We have shown previously that protein and mRNA for PPARγ are expressed in ovarian tissue from immature, untreated rats by 23 days of age [28,31,32]. To determine when the expression of PPARγ is initiated in the rat, ovarian tissue was collected from neonatal rats on days 1, 5, 7, 11, 14, and 19 pp (day 0 = day of birth). These time points correspond to the development of primordial (day 1), primary (days 5, 7), secondary (days 7, 11), and antral (days 14, 19) follicles [36]. Additionally, we also assessed the role of FSH in initiating the expression of PPARγ because the expression of this transcription factor is localized to granulosa cells of developing follicles [28,32].

**Figure 1:**
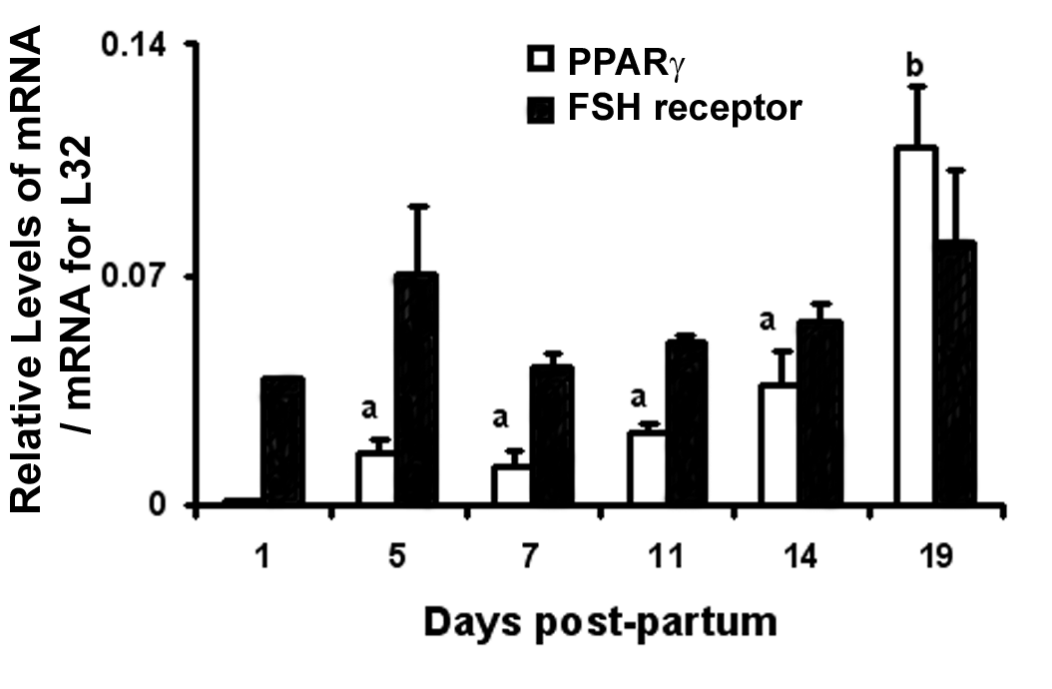
**Relative levels of mRNA for PPARγ and the FSH receptor in ovaries collected from neonatal rats 1 (n = 2), 5, 7, 11, 14, and 19 days pp (n = 3)**. Tissues were collected and processed as described in the Methods. Data are presented as means ± SEM. Bars with no common superscripts are significantly different (p < 0.05).

**Figure 2:**
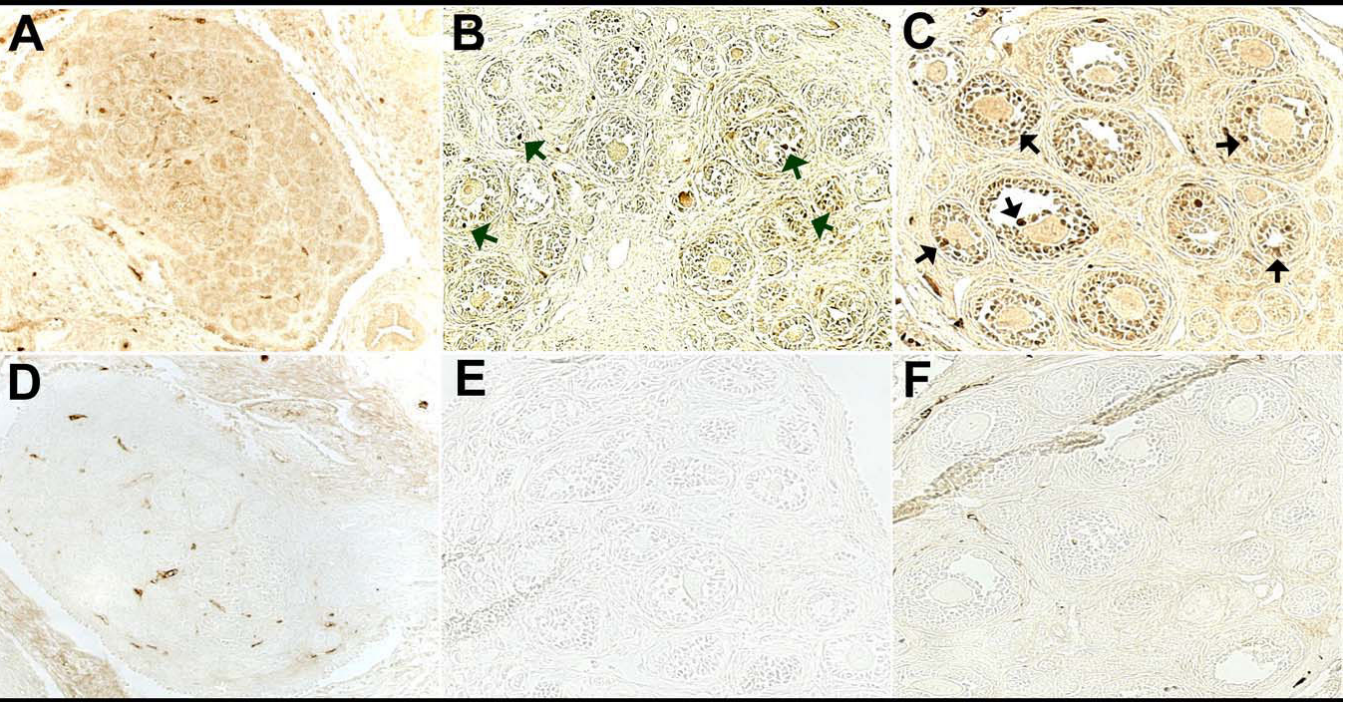
**Immunolocalization of PPARγ in ovarian tissue sections collected from neonatal rats day 5 (A, D), 7 (B, E), and 11 (C, F) pp (n = 3-4 animals/time point)**. Tissues were collected and processed as described in the Methods. PPARγ is identified by the brown reaction product. Tissues in D, E, and F were processed with normal goat serum in place of the anti-PPARγ antibody. Arrows in B and C indicate granulosa cells expressing high levels of PPARγ. Original magnification = 200×.

**Figure 3:**
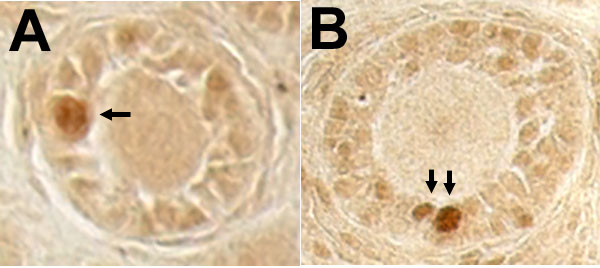
**Immunolocalization of PPARγ in select cells of primary (A) and primary/secondary (B) follicles in ovarian tissue sections collected from neonatal rats**. Tissues were collected and processed as described in the Methods. PPARγ is identified by the brown reaction product. Arrows indicate granulosa cells expressing high levels of PPARγ. Original magnification = 200×.

**Figure 4:**
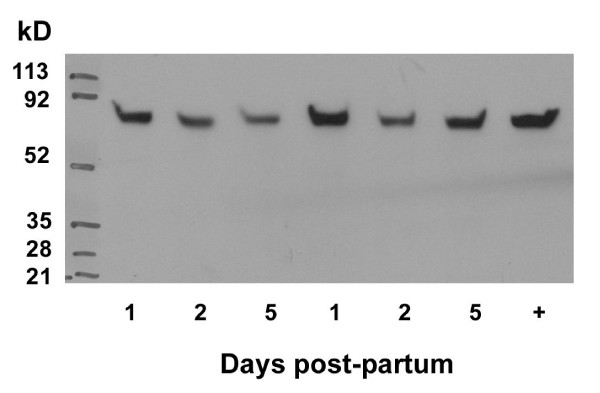
**Western blotting of FSH receptor in ovarian tissue from neonatal rats**. Ovaries were collected at defined times post-partum and processed as described in the Methods (n = 3-4 animals/day). Each lane represents and individual animal. "+" - positive control: ovarian tissue collected 48 hours post-PMSG.

Messenger RNA for PPARγ was not measurable until day 5 pp and levels remained low until the later stages of the neonatal period when they increased between days 14 and 19 pp (Figure 1; p < 0.05). PPARγ was first identified in neonatal rat ovaries collected on day 7 pp (Figure 2). The expression of PPARγ was localized to granulosa cells of primary/secondary follicles (Figure 3), and its expression increased as follicular development progressed (Figure 2). Expression of the FSH receptor was determined in relationship to the expression of PPARγ during the neonatal period. Messenger RNA for the FSH receptor was identified as early as day 1 pp and levels remained relatively steady through day 19 pp (Figure 1). The FSH receptor was detected as early as day 1 pp (Figure 4) and was identified at all time points examined. Detection of actin in these samples denoted equal loading of protein per lane (data not shown).

**Figure 5:**
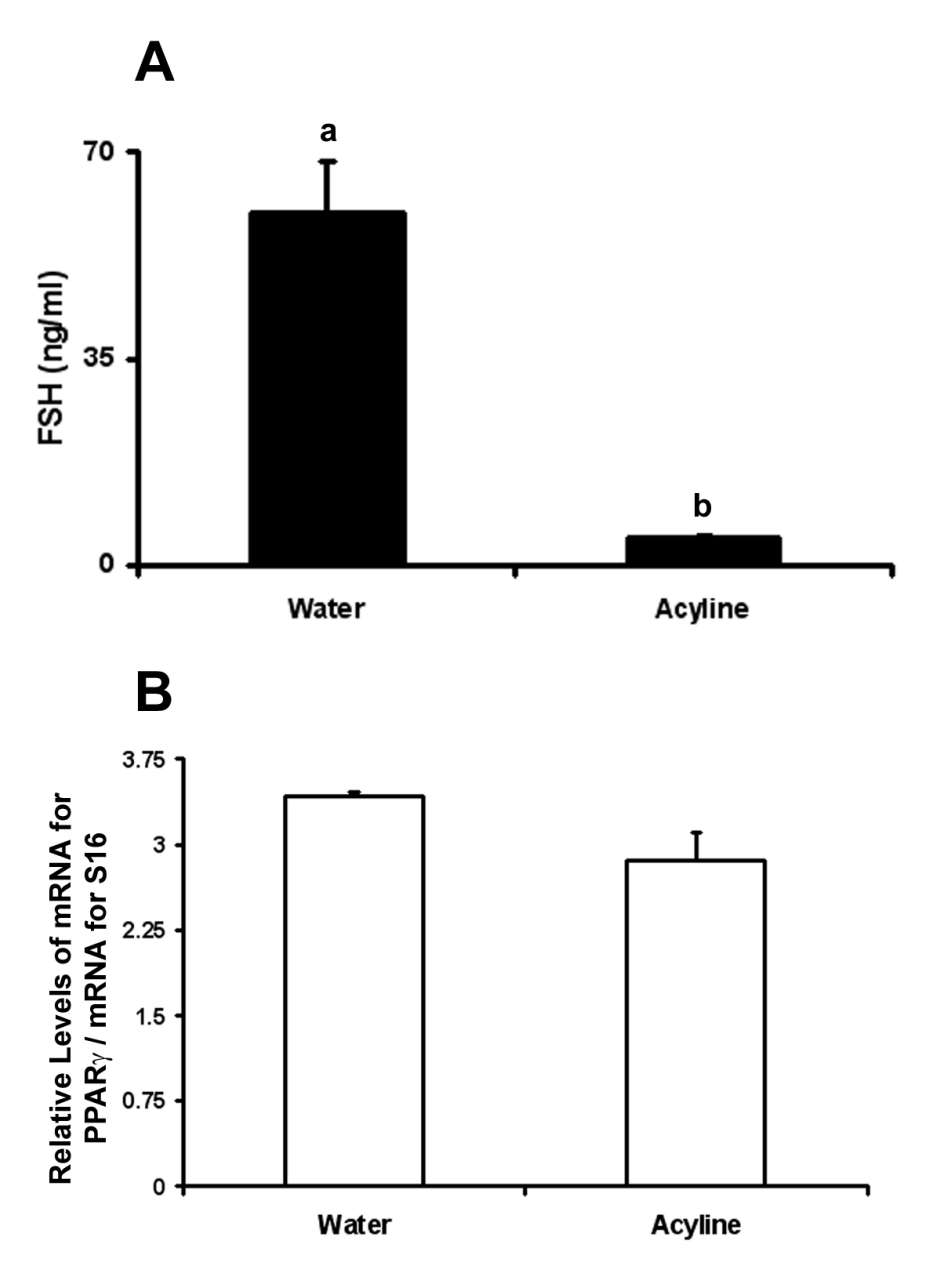
**Circulating concentrations of FSH (A) and relative levels of mRNA for PPARγ in ovarian tissue (B) collected from neonatal rats on day 8 pp (n = 7 in control group; n = 6 in acyline group)**. Data are presented as means ± SEM. Bars with no common superscripts are significantly different (p < 0.05).

Seeing that both mRNA and protein for the FSH receptor were present in ovarian tissue prior to that of PPARγ, the role of FSH in initiating expression of PPARγ was examined. Neonatal rats were treated with the GnRH antagonist, acyline. The concentration of FSH was significantly reduced in animals treated with acyline compared with controls (Figure 5A; p < 0.001). However, levels of mRNA for PPARγ in ovarian tissue collected from treated and control animals were not different (Figure 5B; p = 0.06).

**Figure 6:**
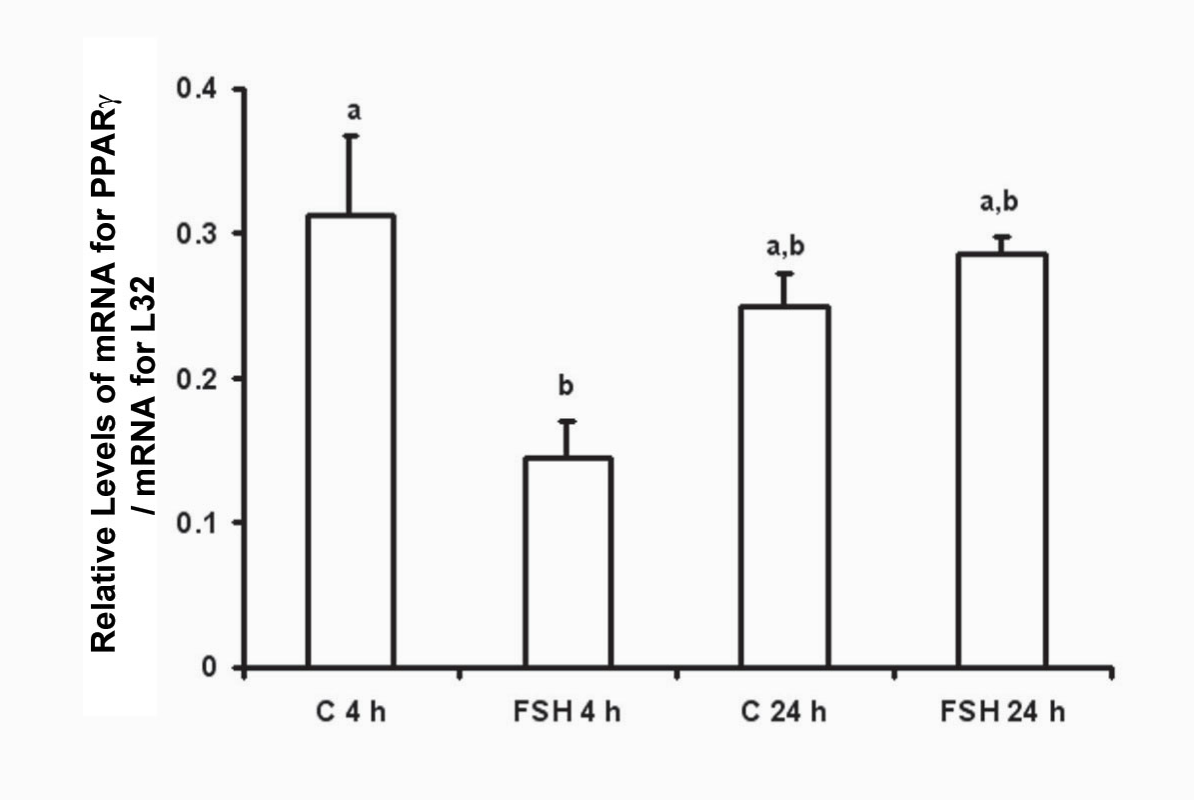
**Relative levels of mRNA for PPARγ in rat granulosa cells cultured *in vitro *as described in the Methods for 4 or 24 hours (h) (n = 3 independent experiments)**. Data are presented as means ± SEM. Bars with no common superscripts are significantly different (p < 0.05). C = control; FSH = 50 ng/ml.

Further assessment of the effect of FSH on the expression of PPARγ was done using cultured granulosa cells collected from estradiol-primed juvenile rats. After 4 hours of culture, levels of mRNA for PPARγ were lower in cells treated with FSH (50 ng/ml) compared to controls (Figure 6; p < 0.05). In contrast, levels of mRNA for PPARγ were not different between cells cultured with or without FSH for 24 hours (Figure 6; p > 0.05). There also was no difference between levels of mRNA for PPARγ between cells cultured for 4 or 24 hours, with or without FSH (Figure 6; p > 0.05).

**Figure 7:**
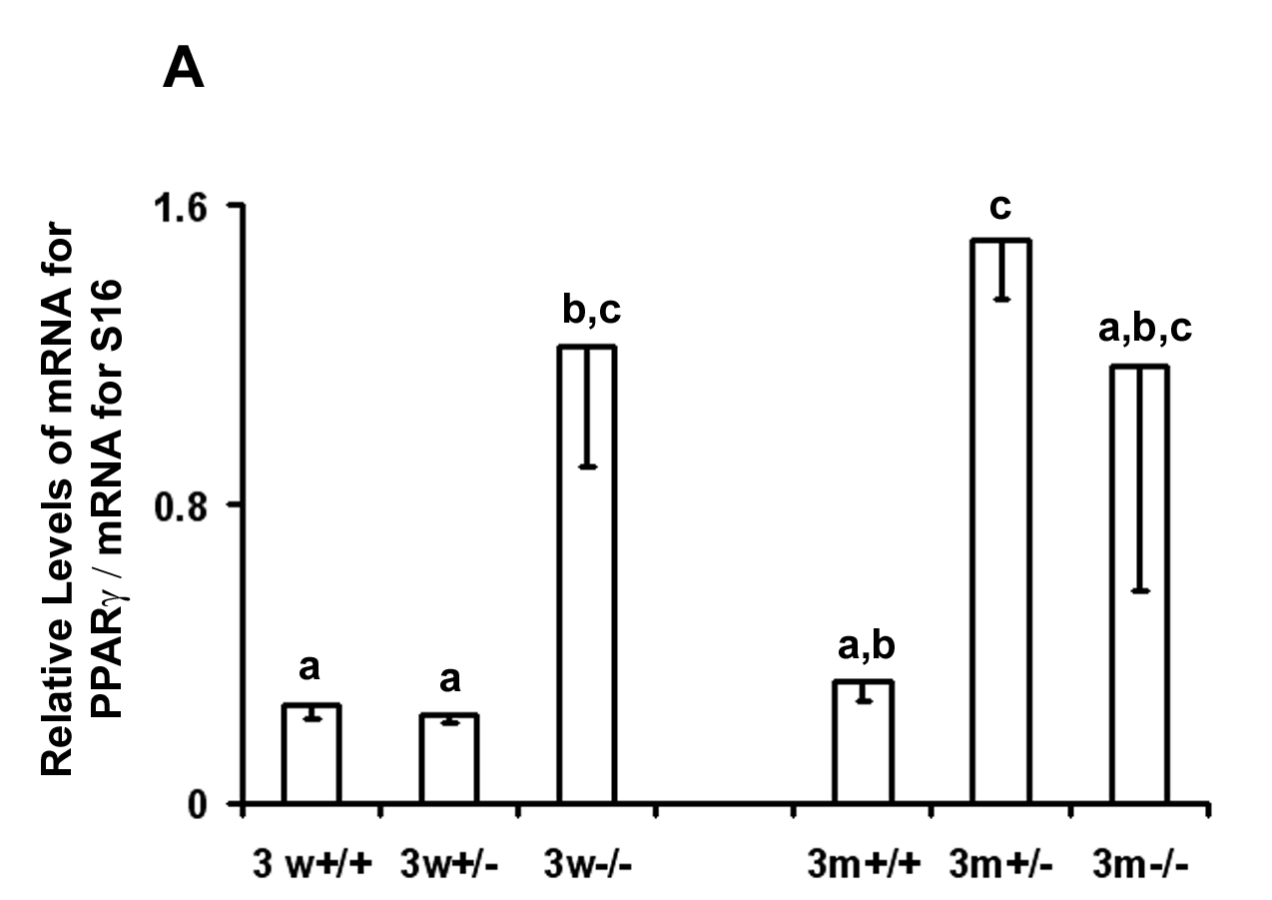
**Relative levels of mRNA for PPARγ in ovarian tissue collected from FORKO mice**. Tissues were collected and processed as described in the Methods from animals with two (+/+), one (+/-) or no (-/-) alleles for the FSH receptor. Animals were aged either 3 weeks (3 w) or 3 months (3 m) when tissues were collected (n = 3 animals/genotype/age group). Data are presented as means ± SEM. Bars with no common superscripts are significantly different (p < 0.05).

Ovarian tissue from FORKO mice was analyzed to determine how the inability to respond to FSH would affect the expression of PPARγ in the ovary. Variable expression of PPARγ was noted in tissues from immature (3 weeks old) and adult (3 months old) females (Figure 7). Levels of mRNA for PPARγ were higher in homozygous knockout immature animals compared to immature heterozygous and wild-type females (p < 0.05), but were not different from adult animals (Figure 7). Expression of mRNA for PPARγ in adults was only different between the wild-type and heterozygous females. PPARγ was detected in tissues from all animals in both age groups (data not shown).

## Discussion

Results from a study by Cui *et al*. [29] indicate that PPARγ plays an important role in normal ovarian function. Using *cre/loxP *technology, the expression of PPARγ was disrupted in numerous tissues including the ovary, rendering 1/3 of the female mice sterile and the remaining females subfertile [29]. Because the expression of PPARγ was not disrupted in the uterus of these transgenic females the authors concluded that "...ovarian function might not be sufficient to induce implantation" [29]. A recent study by Kim *et al*. 2008 also implicates PPARγ in the process of ovulation in mice [37].

The studies described herein are the first, to the best of our knowledge, to investigate when the expression of PPARγ is initiated in the rat ovary. The data presented demonstrate that the expression of PPARγ commences in select, but not all, granulosa cells as early as the primary/secondary stage of development. As follicles continue to develop the number of granulosa cells expressing PPARγ increases. The early stages of follicular development involve long, temporal processes [38]. The progressive development of PPARγ expression may reflect an advancement of gene expression patterns associated with the maturation of granulosa cells. These data indicate that PPARγ may be acting as a regulator of follicular development.

The observed onset of detection of PPARγ in granulosa cells suggested that its expression might be regulated by FSH. During the neonatal period, concentrations of FSH in serum are very high relative to those in immature, juvenile rats [39]. Detection of mRNA for the FSH receptor has been reported as early as day 2 post-partum, the earliest time point investigated, in rat ovaries [40]. Receptors for FSH were detected on type 2 follicles, those with 1-2 layers of granulosa cells ([41]; reviewed in [42]). Binding of FSH to its receptor occurs as early as day 3 post-partum (the earliest time point investigated; [43]). Our data illustrating the detection of mRNA and protein corresponding to the FSH receptor as early as day 1 post-partum are in line with these earlier observations. Since mRNA and protein for PPARγ were not measurable by the methods used in these studies until days 5 and 7 respectively, taken together, these results support the conclusion that the FSH/FSH receptor system is present in neonatal rat ovaries prior to the onset of PPARγ expression.

Despite the temporal relationship between the expression of FSH/FSH receptor and PPARγ in the ovary, reducing circulating concentrations of FSH by 92% following GnRH antagonist treatment did not alter the expression of mRNA for PPARγ. Although not measured in our study, we assume that LH was also reduced but this did not impact PPARγ expression. In addition, although there was a significant decrease in levels of mRNA for PPARγ in cultured granulosa cells 4 hours after treatment with FSH, levels recovered and after 24 hours were not different from control levels after 4 hours of culture. The reason for the initial decrease in mRNA for PPARγ is uncertain, but may reflect an acute response to the relatively high dose of FSH administered. Although not as well understood as signaling mechanisms for LH, the binding of FSH to its receptor activates numerous second messenger systems directly and/or indirectly (reviewed by [44]). The acute accumulation of one or more products (i. e. cAMP; IP_3_) may have caused a reduction in mRNA for PPARγ-mimicking effects of high doses of LH on PPARγ [28]. Catabolism of that second messenger as time in culture progressed may have allowed for the concentration of mRNA for PPARγ to return to levels not different from untreated controls.

The varied expression of PPARγ in the ovaries of FORKO animals also suggests that the expression of PPARγ is not under the primary control of exposure to FSH. Because the expression of PPARγ in heterozygous knockout animals in relationship to homozygous knockout or wildtype animals varied with age, it might be concluded that ovarian morphology and/or endocrine environment are more critical players affecting the expression of PPARγ. Ovarian morphology in FORKO animals is abnormal as early as day 2 post-partum [45]. The altered population of follicles at various stages of development in these animals, and the fact that when adult they do not cycle, may be the reason for the varied concentrations of mRNA for PPARγ due to its expression being associated with progression of follicular development. Also, at 24 days of age estradiol is undetectable in FORKO mice, whereas testosterone and LH are significantly elevated [45]. The altered endocrine environment in FORKO females may have affected the expression of PPARγ. Of interest is the observation that although not always significant, there was a trend for higher levels of mRNA for PPARγ in ovaries from animals with reduced concentrations of estradiol (heterozygous and homozyous knockout animals), especially at 3 months of age. Studies are currently underway to investigate the impact of estradiol on the expression of PPARγ and how it, in relation to other hormones (i. e. testosterone and progesterone), is associated with PPARγ.

The data presented from the current study agree with our previous work investigating PPARγ during the periovulatory period. Its expression is relatively high in granulosa cells prior to the developmental point when granulosa cells express the LH receptor [31]. As expression of the LH receptor increases, the relative expression of PPARγ decreases, and is dramatically reduced in response to the LH surge [28,31]. Therefore, it appears that the primary role of PPARγ is during the early stages of follicular development. Although its expression is down-regulated by one gonadotropin, LH, it does not appear that FSH is a primary player initiating its expression.

PPARγ is functional in the ovary. It binds DNA in rat granulosa cells [46] and results from studies transiently transfecting granulosa cells with reporter constructs demonstrated activity both in the absence and presence of exogenous agonists [24,47]. These latter findings suggest that endogenous agonists of PPARγ are also present and active in granulosa cells. The studies presented herein show that PPARγ expression is initiated very early during folliculogenesis and that expression is associated with the progressive maturation of granulosa cells. Although our hypothesis that FSH regulated the expression of PPARγ was not supported, the findings from these studies indicate that other factors such as estradiol may play an important role. Because agonists of PPARγ have been shown to restore ovulation in some women with PCOS [14] and can improve oocyte quality in mice [48], it is important to understand how this transcription factor is regulated in the ovary.

## Competing interests

The authors declare that they have no competing interests.

## Authors' contributions

MJL carried out all experiments involving rats and assisted in study design. MRS was responsible for studies involving FORKO mice and participated in drafting the manuscript. CMK conceived of the study, participated in study design, coordinated experiments, and drafted the manuscript. All authors read and approved the final manuscript.
